# Captains vs. All-Stars: Who makes better leaders?

**DOI:** 10.1371/journal.pone.0309374

**Published:** 2024-11-14

**Authors:** Alperen Kocsoy

**Affiliations:** Department of Economics, Durham University, Durham, Durham, United Kingdom; Universita degli Studi di Siena, ITALY

## Abstract

This study explores the impact of formally assigned leaders (team captains) and informal leaders (all-stars) on their teammates’ productivity in basketball. It uses in-game injuries as random shocks to examine how the unexpected absence of leaders affects team and individual performance. The research employs a staggered difference-in-differences estimation, to study peer effects in high-stakes team environments. The key finding is that only players who are both formal and informal leaders have spillover effects on their teammates’ performance. The findings could extend to team management practices across various industries, providing insights into effective team composition and leader selection.

## 1 Introduction

Are workers paid according to their marginal productivity? The classic economic model claims that workers are paid according to their marginal productivity of labour. However, in team environments, this becomes more complex as workers contribute not only directly, but also indirectly by influencing the productivity of their teammates and colleagues [1]. Some workers may be more talented in terms of influencing and leading others using their knowledge or charisma [2]. However, it is not always easy to understand the contribution of individuals to team production in many industries because of the scarcity of data. Team sports can offer invaluable insights about the effect of peers on individual and team productivity to understand such dynamics as sports competitions provide team and individual-level time-varying observable productivity data.

Peer effects have been analysed in different areas ranging from academia to workplaces. In economics, studies have shown the complex ways in which individuals influence each other’s productivity and behaviour across diverse settings. For example, [3] find evidence of positive spillovers from high-productivity workers to their peers in a large supermarket chain. Similarly, [4] observe that workers are more productive when working alongside their more able colleagues in a fruit-picking farm. In academia, [5] demonstrate significant decreases in output after the unexpected death of superstar researchers, highlighting the important role of superstars in fostering high productivity and innovation.

In sports, the concept of peer effects has been extensively studied, offering valuable insights due to the availability of observational data and the high-stakes context of professional sports. Studies across various sports disciplines have shown how the presence and performance of peers can significantly affect individual and team productivity. For instance, in basketball, [1, 6] emphasise the positive spillovers from high-performing players to their teammates in the NBA. In golf, [7] highlights how the withdrawal of a superstar competitor can motivate remaining players to perform better.

However, studies find contradictory results. While some find a positive effect, others find negative or no significant effects. Such differences have been explained by the different mechanisms in the contexts of the studies. For example, [8] finds no significant peer effects in golf, suggesting that peer effects may be limited in individualistic sports. This diversity in findings underscores the complexity of peer effects and their dependence on specific contexts and conditions.

On the other side, hiring superstars and selecting team captains are important decisions for sports teams as most of the time such kinds of players are seen as leaders who can increase the performance of their teammates. Superstars, known for their exceptional skills and marketability, are usually paid high salaries, raising questions about their effect on their teammates. It is believed that thanks to their talent, knowledge and charisma, they can be a source of spillover or convince others to perform better. Moreover, team captains are expected to lead and motivate to enhance the team performance. However, the efficacy of team captains and their impact on team success have not been empirically explored, yet.

The effect of superstars in sports has been analysed empirically on various aspects. Studies have examined the impact of superstars on peer performance, showing that their presence can motivate [7, 9] or demotivate [10] competitors, and have positive spillover effects on teammates [6]. Superstars also influence economic factors such as stadium attendance [11–16], attendance at away games [17], and TV audience ratings [18, 19].

In contrast to the extensive research on superstars, the empirical research on the contribution of team captains to their teams is limited. To date, only sports psychologists have studied the characteristics and importance of team captains using qualitative research techniques [20–22]. These studies have challenged the traditional belief that team captains are always the most effective leaders, suggesting that the optimal leadership structure within sports teams may not always align with formal roles. This gap in the literature calls for a more rigorous empirical investigation of the impact of team captains on team performance.

In this paper, I investigate the peer effect of formally assigned leaders (team captains) and informal leaders (All-Stars) by using in-game injuries and foul-outs as random exogenous shocks [6] in staggered difference-in-differences estimation [23]. It is important to note that this study specifically focuses on on-court leadership, i.e., the impact of leaders while they are actively playing. While I acknowledge that players may continue to provide some leadership from the bench when injured or fouled out, the study centres on the immediate effects of their absence from active play.

When discussing leadership in sports teams, many people often think of team managers or head coaches. However, there can also be leaders among athletes and players themselves [20]. Although athlete leadership is a very popular topic among sports psychologists, empirical research on the effects of athlete leaders on their teammates is scarce. This gap is particularly pronounced in understanding how formal leadership roles, such as team captaincy, interact with the informal influence of talented players, who could be All-Star players. The study aims to bridge this gap by offering new insights into how different forms of leadership roles in high-stakes team environments. For the purposes of this study, I briefly define All-Stars as players selected for the NBA All-Star game in the current or previous season, and team captains as players formally designated as captains by their teams. More detailed definitions and discussions of these groups will be provided in the methodology section.

This study combines a methodological approach using in-game injuries and foul-outs (in the NBA, a player is disqualified from the game after accumulating six personal fouls) as unexpected and exogenous shocks, as in [6], providing a natural experiment setting to observe the impact of superstars and team captains on other players in real-time using play-by-play game data. This method allows me to isolate the immediate effects of such players’ absence. This study will help us understand if hiring a superstar affects the performance of incumbents in a team of workers. On the other side, are team captains able to help their team members to perform better? If such a thing is possible, are teams able to choose their team captains effectively?

The implications of the findings extend beyond the scope of professional sports, as they may offer insights for team management across different industries. Understanding the effects of leadership can inform strategies for team composition, leader selection, and performance optimisation. For sports teams, this could translate into more informed decisions regarding captain selections and talent acquisitions. In other corporate environments, these insights could guide leadership development and team-building strategies.

The remainder of this paper is organised as follows: Section 2 presents the data and summary statistics. Section 3 outlines the empirical strategy while Section 4 presents the results. The paper concludes with Section 5, which summarises the key findings and implications of the study for sports teams and general team environments.

## 2 Data and summary statistics

This study utilises data from NBA seasons from 2002 to 2021. The primary sources of data are:

- Play-by-play data from ESPN
- Injury data from Pro Sports Transactions
- Running lists of active playing players from Basketball-Reference
- ELO ratings from FiveThirtyEight

In basketball, a ‘play’ refers to a single action or sequence of actions that occurs during the game. This could be a shot attempt, a pass, a rebound, a turnover, a foul, or any other discrete event that impacts the course of the game. Play-by-play data provide a detailed, chronological record of every such play that occurs during a basketball game. This includes information on scoring plays, rebounds, assists, turnovers, fouls, and substitutions, all timestamped to show exactly when each event occurred. Running lists of active playing players, on the other hand, track which players are on the court at any given moment during the game. These lists are updated in real-time as substitutions occur, allowing for precise analysis of player combinations and their impact on the game. Together, these data sources offer a comprehensive view of game dynamics, player performance, and team strategies as they unfold throughout each match.

ELO ratings, originally developed for chess rankings, are a method of calculating the relative skill levels of players in competitor-versus-competitor games. In the context of the NBA, ELO ratings provide a measure of team strength that takes into account factors such as win/loss record, margin of victory, and strength of schedule. A higher ELO rating indicates a stronger team, with the rating updated after each game based on the result and the ratings of the competing teams.

In this study, I categorise players into three different leadership groups:

- All-Stars only: Players selected for the NBA All-Star game in the current or previous season, but not designated as team captains.
- Team captains only: Players formally designated as captains by their teams for the season, but not selected as All-Stars.
- Both All-Stars and team captains: Players who meet both criteria, are selected as All-Stars and designated as team captains.

This categorisation allows for a detailed analysis of how different forms of leadership—informal talent-based (All-Stars), formally assigned (captains), and the combination of both—impact team productivity and individual performance. The All-Star status serves as a proxy for high skill level and potential informal leadership, while the captain designation represents formal leadership roles within the team.

For robustness, I extended the timeframe for All-Star status by considering players chosen up to 5 years ago as leaders and obtained similar results. It’s worth noting that players missing All-Star games due to prolonged injuries may lead teams to develop new strategies, potentially building resilience to their absence. This study focuses on the unexpected absence of leaders, which aligns with its primary objective of examining their immediate impacts.

To identify in-game injuries, I employ a technique similar to [6]. First, I identify games where leaders were absent due to an injury. Then, I examine the last game they played before being reported as injured. If a leader player leaves the game before the end of the third quarter and does not play again, I record it as an in-game injury. I also gradually relaxed this assumption to include players leaving games until the last two minutes, and the findings remain robust.

Table 1 presents the descriptive statistics of the player-match level data, while Table 2 shows the descriptive statistics of the play-by-play data and S4 Table in S1 File shows the distribution of observations before and after the treatments by leader groups.

**Table 1:** Descriptive statistics of player-match level data.

	N	Mean	St. Dev.	Min	Max
**Game-Related Statistics**					
Season	674,837	2,011.418	5.617	2,002	2,021
Playoff (1 if a playoff game)	674,837	0.066	0.248	0	1
Home Game (1 if played at home)	674,837	0.000	0.000	0	0
Team Elo Rating	674,490	1,510.981	109.748	1,155.440	1,865.449
Team Payroll (in nominal USD)	674,554	82,235,975	27,911,210	33,458,932	178,980,766
Total Match Score	674,837	100.874	13.270	53	196
**Player-Related Statistics**					
All-Star (1 if played in All-Star game)	667,132	0.148	0.355	0	1
Captain (1 if player is team captain)	667,132	0.195	0.396	0	1
Both (Captain and All-Star)	667,132	0.101	0.301	0	1
Captain Only	667,132	0.095	0.293	0	1
All-Star Only	667,132	0.048	0.213	0	1
Draft Number	550,439	20.877	15.109	1	75
Salary (in nominal USD)	459,527	5,486,141	6,237,637	0	43,006,362
Age	643,362	27.138	4.292	18	45
Experience in NBA (in years)	643,293	5.935	4.084	0	23
**Player-Game Statistics**					
Minutes Played in Game	527,015	23.464	11.409	0	65
Real Plus-Minus (RPM)	344,855	0.000	10.740	−60	59
Field Goals Made	527,015	3.643	3.067	0	28
Field Goals Attempted	527,015	8.029	5.767	0	50
3-Point Field Goals Made	527,015	0.741	1.202	0	14
3-Point Field Goals Attempted	527,015	2.078	2.519	0	24
Free Throws Made	527,015	1.763	2.384	0	26
Free Throws Attempted	527,015	2.322	2.925	0	39
Offensive Rebounds	527,015	1.063	1.427	0	18
Defensive Rebounds	527,015	3.067	2.714	0	25
Rebounds	527,015	4.130	3.535	0	31
Assists	527,015	2.133	2.507	0	25
Steals	527,015	0.733	0.985	0	10
Blocks	527,015	0.473	0.884	0	12
Turnovers	527,015	1.332	1.408	0	12
Fouls	527,015	2.033	1.508	0	6
Points	527,015	9.790	8.157	0	81
Starter (1 if player was in starting 5)	674,837	0.375	0.484	0	1
Did not Play (1 if player did not play)	674,837	0.128	0.334	0	1

Note: Player-Match level dataset.

**Table 2:** Play-by-play descriptive statistics.

Statistic	N	Mean	St. Dev.	Min	Max
**Game-Related Statistics**					
Home (1 if game played at home)	10,758,743	0.502	0.500	0	1
Playoff (1 if a playoff game)	10,758,743	0.066	0.249	0	1
All-Star (1 if played in all-star game)	10,758,743	0.232	0.422	0	1
Captain (1 if team captain)	10,758,743	0.291	0.454	0	1
Both (1 if both captain and all-star)	10,758,743	0.178	0.382	0	1
Home Score	10,758,743	52.884	30.999	0	168
Away Score	10,758,743	51.153	30.230	0	168
Elo Rating	10,754,902	1511.127	110.130	1155.440	1865.449
Team Payroll (in USD)	10,758,743	84,282,942	28,490,468	33,458,932	178,980,766
Scoreline (score difference at point)	10,758,743	1.731	10.432	−78	78
**Player-Related Statistics**					
Player Salary (in USD)	7,081,047	7,886,410	7,610,556	0	43,006,362
Age	7,600,027	26.654	4.103	18	44
Experience (in years)	7,593,696	6.028	3.995	0	23
Draft Number	6,901,023	18.006	14.612	1	75
**Play-Related Statistics**					
Scoring Play (1 if scored)	10,758,743	0.251	0.433	0	1
Score Value	10,758,743	0.425	0.850	0	3
Shooting Play (1 if a shot attempted)	10,758,743	0.480	0.500	0	1
Half	10,758,743	1.509	0.500	1	2
Period/Quarter	10,758,743	2.549	1.139	1	8
Distance (in feet)	10,758,743	16.753	10.263	0	93
Cumulative Fouls	10,044,537	1.119	1.180	0	6
**Play-Related Dummies**					
Free Throw	10,758,743	0.015	0.120	0	1
Two Point	10,758,743	0.233	0.423	0	1
Three Point	10,758,743	0.003	0.053	0	1
Foul	10,758,743	0.093	0.291	0	1
Ejection	10,758,743	0.00001	0.003	0	1
Turnover	10,758,743	0.060	0.237	0	1
Rebound	10,758,743	0.229	0.420	0	1
Dunk	10,758,743	0.016	0.126	0	1
Layup	10,758,743	0.080	0.271	0	1
**Absence-Related Statistics**					
Allstar Injury Dropout	10,758,743	0.004	0.066	0	1
Captain Injury Dropout	10,758,743	0.006	0.079	0	1
Allstar 6^th^ Foul	10,758,743	0.0002	0.015	0	1
Captain 6^th^ Foul	10,758,743	0.0003	0.017	0	1

Note: Play-by-play level dataset.

Table 3 descriptively shows mean salary and Real Plus-Minus (RPM) by player roles. I use the salaries of players as a proxy for the talent they have. I use nominal values of salaries as I include team-season fixed effects which capture the impact of inflation.

**Table 3:** Salary, RPM, Minutes, and RPM per Minute by player role.

Role	Mean Salary	Mean RPM	Mean Minutes	RPM per Minute
Regular Player	3,995,778	-0.3361376	20.65859	-0.0780085
Captain Only	9,425,869	-0.0220852	27.83307	-0.0295563
Allstar Only	8,688,743	1.4186897	26.65745	0.0185244
Both	16,943,390	2.5865464	33.42574	0.0752740

Note: RPM: Real Plus-Minus. Salary values are in USD.

Basically, Plus-Minus measures the net point difference when a player is on the court, providing a metric of the contribution of players to the result while actively playing. However, this metric neglects the quality of teammates and opponents. To address this, Adjusted Plus-Minus was developed by employing statistical models to refine Plus-Minus data by considering the varying qualities of teammates and opponents, aiming to isolate an individual player’s contribution more accurately but neglecting the overall quality of the player. Building on this, Real Plus-Minus (RPM), developed by analysts at ESPN, incorporates additional player statistics and more sophisticated adjustments for team dynamics and opposition quality, offering a comprehensive metric that captures a player’s overall impact with greater precision [24].

Unfortunately, Real Plus-Minus (RPM) data are not provided for play-by-play data. To be able to capture the team productivity and individual contribution before and after the treatment, I trained long short-term memory (LSTM) networks using the historical performance of players embedded in the play-by-play and end-of-game RPM of players. LSTM networks are a kind of recurrent neural network system designed to learn from sequences of data by capturing important patterns over long intervals and are highly used to predict data based on time series [25]. The model predicts the RPM of players at every point and shows any changes from the previous one to the next in the play-by-play data in sequential order. Then, I validated the end-of-game RPM values of the trained model with that of ESPN. S3 Fig in S1 File shows the scatter plot of predicted and ESPN RPM in the Supporting Information.

## 3 Empirical strategy

In this part, I begin with a preliminary analysis to see if leader players (captain and All-Star) are better performers than non-leaders as there could be a potential spillover of performance which can enhance the performance of non-leaders. Team captains are formal leaders of teams assigned by coaches or team managers. On the other side, All-Star players are exceptionally talented and played in the All-Star game. The All-Star game is a single game that is a showcase of talent in the NBA as the most talented players are chosen by experts and public votes every year. Some players are chosen thanks to their exceptional talent while others thanks to their popularity in the media and public. In that manner, All-Stars can be seen as superstars according to definitions of both [26, 27]. The next part compares the performance of player types before the examination if such players can affect the performance of their teammates.

### Leaders vs. Others

To test if leader players perform better than others, I use the OLS regression equation given below with the player-match level dataset. The main dependent variable is Real Plus-Minus (RPM) while other boxscore metrics are also used to understand in-game dynamics.

$$\begin{array}{ccc} {\text{Performance}}_{imst} & = & \beta_{0}+\beta_{1}\times {\text{Both}}_{imst}+\beta_{2}\times \text{Captain}\;{\text{Only}}_{imst}\\ & & +\beta_{3}\times \text{All-Star}\;{\text{Only}}_{imst}+\gamma \times {\text{Controls}}_{imst}\\ & & +\alpha_{\text{Team}\times \text{Season}}jt+\alpha_{\text{Opponent}\times \text{Season}}kt\\ & & +\alpha_{\text{Player}\;\text{Position},i}+\varepsilon_{imst} \end{array}$$ (1)

Where:

- Performance_*imst*_ represents the productivity of player *i* in match *m* during season *s* at time *t*.
- Both_*imst*_, Captain Only_*imst*_, and All- Star Only_*imst*_ are dummy variables indicating the leadership status of player *i*: being both a captain and an All-Star, only a captain, or only an All-Star, respectively, in match *m* during season *s* at time *t*.
- **γ** × Controls_*imst*_ includes control variables such as minutes played, player’s age, and other game-specific factors that might influence a player’s productivity.
-
$\alpha_{\text{Team}\times \text{Season}}jt$ and $\alpha_{\text{Opponent}\times \text{Season}}kt$ are fixed effects that account for team-season and opponent-season interactions, capturing the influence of team dynamics and the competitive environment in each season.
- *α*_Player Position,*i*_ represents fixed effects for the player’s position, controlling for the specific roles and responsibilities associated with each position on the court.
- *ε*_*imst*_ is the error term, capturing unobserved factors affecting player productivity. I assume that the error term is clustered at the player level to account for potential correlations within players across observations with the presence of fixed effects.

### Absence of leaders

In this main part of the study, I use in-game injuries of leader players as a source for random and unexpected exogenous variation to control their effect on other players [6, 28]. Throughout the games, I keep track of the players who are actively playing on the court and their leadership status (whether they are team captains or All-Stars) using the in-game running lists of players provided for each team. Therefore, I ensure that the injured leader player is substituted with a non-leader to disentangle the effect of leaders on others. Previous studies tried to infer players on the court using event data in the absence of in-game running lists of players and because of that reason needed to drop several observations [1]. Furthermore, I can control for the playing time of players which is claimed endogenous because of the variation in productivity of players in games.

In this part, I examine the effect of absence of leaders in two areas: on performance of other players and on overall team success.

#### Effect of absence of leader on performance of other players

In the main analysis, I use staggered difference-in-differences event study [23], which enables me to detect a precise treatment effect of injuries which take place at different times during the games. Also, the estimation allows me to find the average treatment effect on the treated when the length of exposure to treatment (absence of leader) is different. To analyse the effect of leader absence on the performance and productivity of non-leaders, I use the following model:

$$\begin{array}{ccc} {\text{Performance}}_{itp} & = & \beta_{0}+\beta_{1}\times \text{Leader}\;{\text{Absence}}_{tp}+\gamma \times {\text{Controls}}_{itp}+\alpha_{\text{Player}i}+\alpha_{\text{Game}t}\\ & & +\sum_{j=1}^{3}\alpha_{{\text{Teammate}}_{j}}+\sum_{k=1}^{5}\alpha_{{\text{Opponent}}_{k}}+\varepsilon_{itp} \end{array}$$ (2)

Where:

- Performance_*itp*_ denotes the performance metric of player *i* at play *p* in game *t*, reflecting the real-time productivity of non-leader players.
- LeaderAbsence_*tp*_ is a dummy variable equal to 1 if a leader is suddenly absent (due to injury or fouling out) and replaced by a non-leader in play *p* in game *t* for player *i* and 0 otherwise.
- **γ** × Controls_*itp*_ includes play-specific control variables that might influence performance, such as the current score difference, time remaining in the game, and the quarter.
-
$\alpha_{\text{Player}}i$ represents individual player fixed effects, accounting for unobserved characteristics of player *i* that could affect player productivity.
-
$\alpha_{\text{Game}}t$ captures game-specific fixed effects, reflecting characteristics of game *t* that could affect player productivity.
-
$\sum_{j=1}^{3}\alpha_{{\text{Teammate}}_{j}}$ and $\sum_{k=1}^{5}\alpha_{{\text{Opponent}}_{k}}$ are the sums of fixed effects for the three teammates (except player *i* and leader who will be injured or fouled out) and five opponents on the court, respectively, during play *p*, controlling for the influence of other players type (e.g. any changes in the number of leaders of opponent) in the game.
- *ε*_*itp*_ is the error term for player *i* at play *p* in the game *t*, capturing unobserved factors that might affect performance during that specific play.

#### Effect of absence of leader on team success

To estimate the effect of the absence of leaders on team productivity, I use the following logistic regression model:

$$\begin{array}{ccc} \text{log}(\frac{P({\text{Win}}_{jkt}=1)}{1-P({\text{Win}}_{jkt}=1)}) & = & \beta_{0}+\beta_{1}\times {\text{AllStar}}_{jt}+\beta_{2}\times {\text{Both}}_{jt}+\beta_{3}\times {\text{Captain}}_{jt}\\ & & +\gamma \times {\text{Controls}}_{jkt}+\alpha_{\text{Team}\times \text{Season}jt}+\alpha_{\text{Opponent}\times \text{Season}kt}+\varepsilon_{jkt} \end{array}$$ (3)

Where:

- *P*(Win_*jkt*_ = 1) is the binary outcome of the game between team *j* and opponent *k* at time *t*, where 1 represents a win and 0 a loss.
- AllStar_*jt*_, Both_*jt*_, and Captain_*jt*_ are dummy variables indicating the injury of team *j*’s leaders (All-Star only, both All-Star and Captain or Captain only) at time *t*.
- **γ** × Controls_*jkt*_ represents control variables such as absence leaders of the opponent team, playoff dummy, Elo rating differences, and other factors relevant to the game outcome.
-
$\alpha_{\text{Team}\times {\text{Season}}_{jt}}$ and $\alpha_{\text{Opponent}\times {\text{Season}}_{kt}}$ are fixed effects for interactions between team *j* and season at time *t* and opponent *k* and season at time *t*, respectively.
- *ε*_*jkt*_ is the error term for the game between team *j* and opponent *k* at time *t*.

## 4 Results

### Leaders vs. Others

Table 4 reports the regression results showing if leader type and performance are associated. When the controls and a set of fixed effects are added to absorb or control for unobserved heterogeneity between player positions and time-invariant factors specific to teams, players who are both captains and All-Stars perform better than their teammates significantly and contribute to the team production positively. Conversely, players designated as captains without All-Star experience demonstrate a significant negative performance differential when compared to non-leaders. This may raise a problem with the captain assignment processes in teams. Although such players may still be contributing to the team success in different areas including team cohesion, team captains might be expected to perform well too. On the other side, the performance of All-Star players who have not been assigned as captains shows no significant deviation from that of their non-leader teammates.

**Table 4:** Leader type and performance.

Dependent Variable:	Real Plus-Minus		
Model:	(1)	(2)	(3)
Both (Captain & All-Star)	2.937***	0.5894**	0.4975***
	(0.2603)	(0.1888)	(0.1659)
Captain Only	0.2980	-0.2617*	-0.3795***
	(0.1811)	(0.1309)	(0.1146)
Allstar Only	1.705***	0.2289	0.1063
	(0.3205)	(0.2289)	(0.2007)
Playoff		-0.0773	-0.0490
		(0.0959)	(0.0925)
Minutes		0.1140***	0.1204***
		(0.0036)	(0.0037)
Salary		8.48 × 10^−9^	3.89 × 10^−10^
		(8.32 × 10^−9^)	(8.06 × 10^−9^)
Elo Difference		0.0167***	-0.0035***
		(0.0003)	(0.0004)
Age		0.2583*	0.2502***
		(0.1013)	(0.0918)
Age^2^		-0.0041*	-0.0038**
		(0.0018)	(0.0016)
*Fixed-effects*			
Player Position	No	No	Yes
Team × Season	No	No	Yes
Opponent Team × Season	No	No	Yes
Observations	344,549	288,226	288,226
R^2^	0.008	0.074	0.096

Player-level clustered robust standard errors in parentheses.

* *p* < 0.1;

** *p* < 0.05;

*** *p* < 0.01.

When I examine the sub-metrics of players for performance, I find that captains who played in an All-Star game get more defensive rebounds and steal more balls, suggesting that they spend more effort than others while defending and, meanwhile, surprisingly commit fewer fouls. On the offence, although they lose the ball more than others together with only All-Stars, they assist more than the rest. However, both captains and All-Stars suffer from more fouls as stopping them could be challenging. While all types of leaders attempt more to score, their success rate in three-pointers is slightly lower than their non-leader teammates. On the other side, these players often work longer by staying longer on the court. This could indicate a diminishing rate of marginal productivity, where performance may decline as the game progresses due to fatigue. S1 and S2 Tables in S1 File report regression results of each box score metric.

### Absence of leaders

#### Effect of absence of leader on performance of other players

Table 5 reports the results of canonical difference-in-differences analysis, which estimates the effect of injuries and fouled-outs of leaders on the performance of other players. This 2 × 2 difference-in-difference approach shows that the absence of both(captain and All-Star) due to injuries decreases the performance of other players. The absence of captains or All-Stars and the absence of both due to the 6^th^ fouls do not significantly affect the performance of other players.

**Table 5:** Canonical difference-in-differences results.

Dependent Variable:	*Real Plus-Minus*					
Treatment Reason:	Injury	6^th^ Foul	Injury	6^th^ Foul	Injury	6^th^ Foul
Treatment × *Post: Both*	−0.77(0.31)**	−0.16(0.10)				
Treatment × *Post: Only All − Star*			−0.52(0.31)	−0.11(0.07)		
Treatment × *Post: Only Captain*					−0.30(0.21)	−0.07(0.07)
Play-level Controls	Yes	Yes	Yes	Yes	Yes	Yes
*Fixed-effects*						
Game	Yes	Yes	Yes	Yes	Yes	Yes
Player	Yes	Yes	Yes	Yes	Yes	Yes
Teammates	Yes	Yes	Yes	Yes	Yes	Yes
Opponent Players	Yes	Yes	Yes	Yes	Yes	Yes
Observations	4, 752, 100	4, 752, 100	4, 752, 100	4, 752, 100	4, 752, 100	4, 752, 100
R^2^	0.34	0.34	0.34	0.34	0.34	0.34

Play-level controls include remaining time for the end of the quarter, half-time and match, and scoreline. Two-way (Game & Player-level) clustered robust standard errors in parentheses.

* *p* < 0.1;

** *p* < 0.05;

*** *p* < 0.01.

It is worth noting that while all teams will have a team captain, not all teams will have an All-Star player. However, this difference in the distribution of leadership types across teams does not pose a significant problem for the analysis. The primary reason for this is the use of the difference-in-differences. This approach allows to examine the change in players’ performance before and after the injury of a leader, regardless of the team’s overall composition.

Furthermore, the inclusion of team fixed effects in the models controls for time-invariant characteristics of teams, including whether they typically have All-Stars or not. This means that the analysis effectively compares the performance changes within teams, rather than between teams with different leadership compositions. As such, the potential imbalance in All-Star presence across teams is accounted for in the empirical strategy, ensuring that the findings reflect the true impact of leader absence rather than systematic differences between teams with and without All-Stars.

**Fig 1:**
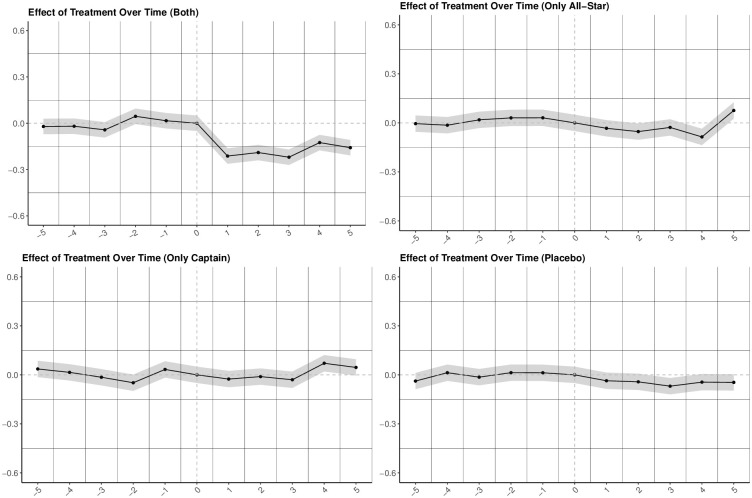
Real Plus-Minus (RPM) of regular players before and after injury of player type. Note: Callaway and Sant’Anna (2021) event study estimations before and after treatments. X-axes: Time to Treatment (in Minutes). The shaded areas represent 95% confidence intervals.

Fig 1 illustrates the RPM of non-leaders before and after the injury of players by the player group along with the placebo leaders. Similar to the findings of canonical difference-in-differences, the result of the event study shows that the RPM of non-leaders is negatively affected by the injury of players who are both team captains and All-Stars. However, injuries of only captains and only All-Star players have no significant effect on the performance of non-leaders.

As shown, team captains who never played in an All-Star game and players who played in an All-Star game but do not have the team captaincy, do not have any effect on the performance of other players. The reason for that could be that they are not better than regular players in teams. This shows that to be able to affect others, leaders need to be a source of a spillover. Some players could be chosen for the All-Star game or assigned as captains because of their popularity. However, the findings show that talent is vital for leading others. A person could be a superstar either through talent [26] and/or through popularity [27]. However, being an effective leader requires superstardom as defined by [26].

This finding aligns with that of [6], which reports a decrease in the field goal percentage of non-high-performing players following the injury of a high-performing teammate. It is conceivable that the high-performing players referred to in their study could correspond to the All-Starred team captains discussed in this analysis as both perform better than other players and have a similar impact on the performance of their teammates and team. However, contrary to [6], my analysis reveals that teams attempt more three-pointers rather than two-pointers in the absence of players who hold both captain and All-Star status. When such leaders are present on the court, 74.4% of shots (combining two and three-point attempts) are two-pointers; this proportion drops to 60.8% in their absence. This shift may derive from the lack of creativity typically contributed by the All-Starred team captain.

Further, by analysing the location and distance of shots, I find that three-point field goals are attempted from greater distances when only All-Starred team captains are absent, whereas the distance of two-point field goals remains unaffected. This could be attributed to the more constrained area for two-pointers and teams opting for two-point shots only when good opportunities arise as the proportion of two-pointer attempts goes down. Also, the effect of the absence of only All-Star players is significant under 10% level showing weak importance of their talent and creativity on the distance of three-pointers. Table 6 below shows the effect of the absence of leaders on the distances of three-pointers using difference-in-differences estimation. S5 Table in S1 File shows a similar table for two-pointers in S1 File.

**Table 6:** Distance of three-point field goal attempts and absence of leaders.

Dependent Variable:	Distance of Three-Point Field Goal Attempts		
Model:	(1)	(2)	(3)
Both: Treatment × Post	0.5580***		
	(0.2098)		
Captain: Treatment × Post		0.3474	
		(0.2294)	
All-Star: Treatment × Post			0.4400*
			(0.2592)
Home	-0.0654***	-0.0657***	-0.0656***
	(0.0094)	(0.0093)	(0.0093)
Score Difference	0.0021***	0.0021***	0.0021***
	(0.0006)	(0.0006)	(0.0006)
Period	-0.0902***	-0.0898***	-0.0888***
	(0.0067)	(0.0067)	(0.0067)
*Fixed-effects*			
Game	Yes	Yes	Yes
Player	Yes	Yes	Yes
Team	Yes	Yes	Yes
Opponent Team	Yes	Yes	Yes
Observations	683,102	683,102	683,102
R^2^	0.110	0.110	0.110

Note: Score Difference is *Team* − *Opponent*. Player-level clustered robust standard errors in parentheses.

* *p* < 0.1;

** *p* < 0.05;

*** *p* < 0.01.

#### Effect of absence of leader on team success

Table 7 reports the logistic regression results of the game result and OLS regression results of score difference at the end of the game with the absence of leader types for home teams only to avoid correlated error terms of duplicate observations. When a player who is a team captain and experienced an All-Star game, is absent, the chance of the team winning the game decreases around 24% (1 − *e*^−0.27^) for home teams and approximately 20% (1 − *e*^−0.22^) for away teams when controls and fixed effects are included. Moreover, in the absence of such players score difference significantly decreases even if teams win the game, meaning teams struggle more. Therefore, team productivity is significantly lower when such key players are missing. For a robustness check, I replicated the analyses using observations of teams that had only one player from any player type and their absence due to injury and found similar results. S6 Table in the S1 File shows the regression results for away teams.

**Table 7:** Score difference, game result and injury of key players (home teams).

Dependent Variables:	Score (1)	Score (2)	Score (3)	Result (1)	Result (2)	Result (3)
Injury of Both	−1.57***	−1.23***	−1.89***	−0.19***	−0.16***	−0.27***
	(0.20)	(0.18)	(0.25)	(0.03)	(0.03)	(0.05)
Injury of Only Captain	−1.59***	−0.30	−0.17	−0.20***	−0.02	0.01
	(0.17)	(0.16)	(0.25)	(0.03)	(0.03)	(0.05)
Injury of Only All-Star	0.15	−0.27	−0.54	0.04	−0.01	−0.08
	(0.19)	(0.17)	(0.28)	(0.03)	(0.03)	(0.05)
Opponent’s Injury of Both	1.20***	0.87***	1.65***	0.13***	0.10**	0.22***
	(0.20)	(0.19)	(0.25)	(0.03)	(0.03)	(0.05)
Opponent’s Injury of Only Captain	1.28***	0.09	0.57**	0.18***	0.01	0.08
	(0.17)	(0.15)	(0.24)	(0.03)	(0.03)	(0.05)
Opponent’s Injury of Only All-Star	−0.64***	−0.17	0.12	−0.07*	−0.00	0.05
	(0.19)	(0.17)	(0.28)	(0.03)	(0.03)	(0.05)
Play-off		1.22***	0.74*		0.11*	0.03
		(0.32)	(0.35)		(0.05)	(0.06)
Elo Difference		0.04***	−0.01***		0.01***	−0.00***
		(0.00)	(0.00)		(0.00)	(0.00)
*Fixed-effects*						
*Team* × *Season*	No	No	Yes	No	No	Yes
*OpponentTeam* × *Season*	No	No	Yes	No	No	Yes
Observations	25562	25549	25549	25562	25549	25549
R^2^	0.01	0.16	0.25			
Deviance				34368.13	31001.53	28727.38
Log Likelihood				-17184.06	-15500.76	-14363.69
Pseudo R^2^				0.01	0.10	0.10

The first three columns show OLS estimations where the dependent variable is the score difference. The remaining three columns show logistic regression where the dependent variable is the match result. Game-level clustered robust standard errors in parentheses.

* *p* < 0.1;

** *p* < 0.05;

*** *p* < 0.01.

## 5 Conclusion

In this study, I examined if the unexpected loss of a talented leader worker affects the productivity of others in teams using data from the NBA. The event study shows that the absence of players who have both captaincy and star-title altogether negatively affects the productivity of individuals and teams while the absence of players assuming only captaincy or star title neither affects the performance of other players nor that of teams.

The findings imply there could be some issues in assigning team captains. Although they may have intangible effects on teams including team cohesion and social leadership, motivating others through performance could be expected too. Teams can benefit from the role of the team captain to identify them as an idol or role model in teams that promote an intrinsic motivation to perform for team identity.

Although being very talented is beneficial for team production, only star players are not able to be a source of spillover and affect others. I conclude that for a peer effect to be significant, certain conditions must be met, such as holding a formal leadership position and possessing superior talent. Without these attributes, the presence or absence of players and managers may not substantially affect the performance of others in teams.

Additionally, by using the distance of shoots, I showed that teams need to take more risks when leaders are absent. That could be the case for employees who need to make decisions affecting their firms and others such as sales and purchasing departments. Managers would have negotiation experience in such transactions and, therefore take fewer risks. However, their absence could be more costly in such operations. This finding shows the importance of both formal and informal leadership roles besides individual skills in driving team success and highlights the complexity of peer dynamics within teams. The study provides implications for people managing teams not only in sports but also in similar environments.

In conclusion, this study contributes to our understanding of leadership in team sports by quantifying the impact of different types of leaders on individual and team performance. It demonstrates that effective leadership in basketball requires a combination of formal recognition (captaincy), high skill levels (All-Star status), and the ability to positively influence teammates’ performance. These findings can inform team management strategies, player development programmes, and tactical approaches in professional basketball and potentially in other team sports and collaborative work environments.

### Limitations and future work

While this study sheds light on the impact of leader absence on team productivity within the context of the NBA, the findings are subject to limitations which could be addressed in future research. The main limitation is the study focuses on a single sport, which may limit the generalisability of the results to other sports or team-based settings. Additionally, the use of NBA play-by-play data, may not be capturing all aspects of leadership and team dynamics that affect the performance of individuals. Future studies could expand this research by replicating the analysis using data from other sports, such as football, hockey, or baseball, to examine whether the observed effects of leader absence hold in different team environments. Moreover, extending the investigation outside of sports, such as teams from other industries, could provide valuable insights into the role of leaders in team productivity. Such cross-disciplinary research could enhance our understanding of how the absence of key individuals affects team outcomes and inform strategies for mitigating these negative effects.

## Supporting information

S1 File Supporting information file has been provided to show the robustness of the findings and the distribution of observations. (PDF)
