# Supplementary material for: Captains vs. All-Stars: Who makes better leaders?
Source: PLoS One. 2024 Nov 14;19(11):e0309374. doi: 10.1371/journal.pone.0309374 (PMC11563377; doi:10.1371/journal.pone.0309374)
Supplement: S1 File — (PDF) [file pone.0309374.s001.pdf]

Captains vs. All-Stars: Who makes better leaders?

Alperen Kocsoy

Supporting Information

S1 Table: Additional Estimations for Leaders and Others

S1 Table: Minutes Played and Scoring Rates in Games

Dependent Variables:	Minutes Played	Free Throw %	Field Goals %	3-Point Field Goal %
Model:	(1)	(2)	(3)	(4)
Both	6.484***	0.0043	-0.0144**	-0.0313***
	(0.7165)	(0.0104)	(0.0051)	(0.0079)
Captain Only	4.557***	-0.0042	-0.0054	-0.0111**
	(0.7986)	(0.0069)	(0.0044)	(0.0033)
All-Star Only	6.223***	-0.0018	-0.0067*	-0.0277***
	(0.4883)	(0.0159)	(0.0032)	(0.0041)
Playoff	0.0584	-0.0117***	-0.0121***	-0.0141***
	(0.2228)	(0.0030)	(0.0012)	(0.0020)
Real Plus Minus	0.0971***			
	(0.0033)			
Salary	5.6×10^{-7}***	8.94×10^{-10}	-7.28×10^{-10}***	-8.48×10^{-10}**
	(4.98×10^{-8})	(7.04×10^{-10})	(1.3×10^{-10})	(2.52×10^{-10})
Elo Difference	-0.0026***	4.11×10^{-6}	-1.55×10^{-5}*	-5.28×10^{-5}***
	(0.0004)	(9.85×10^{-6})	(7.6×10^{-6})	(9.22×10^{-6})
Age	0.8447	0.0143**	0.0010	0.0082**
	(0.5660)	(0.0051)	(0.0024)	(0.0032)
Age^2	-0.0200*	-0.0002**	-2.95×10^{-5}	-9.52×10^{-5}
	(0.0091)	(8.37×10^{-5})	(4.12×10^{-5})	(5.7×10^{-5})
Minutes Played		0.0026***	0.0039***	0.0038***
		(0.0002)	(0.0002)	(0.0002)
Fixed-effects				
Player Position	Yes	Yes	Yes	Yes
Team × Season	Yes	Yes	Yes	Yes
Opponent Team × Season	Yes	Yes	Yes	Yes
Observations	288,226	213,278	346,074	230,452
R^2	0.284	0.0517	0.0608	0.0221

Player-level clustered robust standard errors in parentheses. * $p < 0.1$; ** $p < 0.05$; *** $p < 0.01$.

S2 Table: Additional Estimations for Leaders and Others

S2 Table: Minutes Played and Scoring Rates in Games

Dependent Variables:	Defensive Rebounds	Steals	Fouls Committed	Turnovers	Assists	Fouls Suffered
Model:	(1)	(2)	(3)	(4)	(5)	(6)
Both	0.5855***	0.1253**	-0.1497*	0.5240***	0.8043***	1.934***
	(0.1437)	(0.0391)	(0.0685)	(0.0520)	(0.1798)	(0.1673)
Captain Only	0.1152	0.0430	0.0177	0.1012**	0.0891	0.1976**
	(0.1241)	(0.0315)	(0.0295)	(0.0327)	(0.1199)	(0.0599)
Allstar Only	0.3031**	0.0185	-0.1135*	0.3640***	0.5828**	0.7657***
	(0.1131)	(0.0225)	(0.0505)	(0.0445)	(0.2023)	(0.1160)
Playoff	-0.0250	-0.0495***	0.1777***	-0.0765***	-0.2124***	0.1508***
	(0.0428)	(0.0100)	(0.0112)	(0.0123)	(0.0428)	(0.0181)
Minutes Played	0.1315***	0.0317***	0.0578***	0.0465***	0.0912***	0.0996***
	(0.0165)	(0.0013)	(0.0013)	(0.0030)	(0.0162)	(0.0033)
Salary	2.6×10^{-8}***	-1.67×10^{-9}	-7.67×10^{-9}**	2.46×10^{-8}***	4.17×10^{-8}***	4.21×10^{-8}***
	(4.2×10^{-9})	(1.85×10^{-9})	(3.23×10^{-9})	(3.12×10^{-9})	(8.85×10^{-9})	(7.26×10^{-9})
Elo Difference	-0.0002*	5.47×10^{-6}	-0.0002**	-3.05×10^{-5}*	-0.0003***	0.0002***
	(0.0001)	(3.11×10^{-5})	(6.52×10^{-5})	(1.63×10^{-5})	(8.05×10^{-5})	(5.09×10^{-5})
Age	-0.1865***	0.0136	-0.0214	-0.0704*	0.0425	-0.0156
	(0.0393)	(0.0097)	(0.0157)	(0.0355)	(0.0787)	(0.0356)
Age^2	0.0028***	-0.0003*	0.0003	0.0007	-0.0009	-0.0014*
	(0.0007)	(0.0002)	(0.0003)	(0.0006)	(0.0015)	(0.0006)
Fixed-effects						
Player Position	Yes	Yes	Yes	Yes	Yes	Yes
Team × Season	Yes	Yes	Yes	Yes	Yes	Yes
Opponent Team ×Season	Yes	Yes	Yes	Yes	Yes	Yes
Observations	363,143	363,143	363,143	363,143	363,143	363,143
R^2	0.447	0.166	0.212	0.277	0.448	0.327

Player level clustered robust standard errors in parentheses.* $p < 0.1$; ** $p < 0.05$; *** $p < 0.01$.

S3 Figure: Predicted Real Plus-Minus

S3 Figure: Predicted and ESPN Real Plus-Minus

Predicted and ESPN Real Plus-Minus

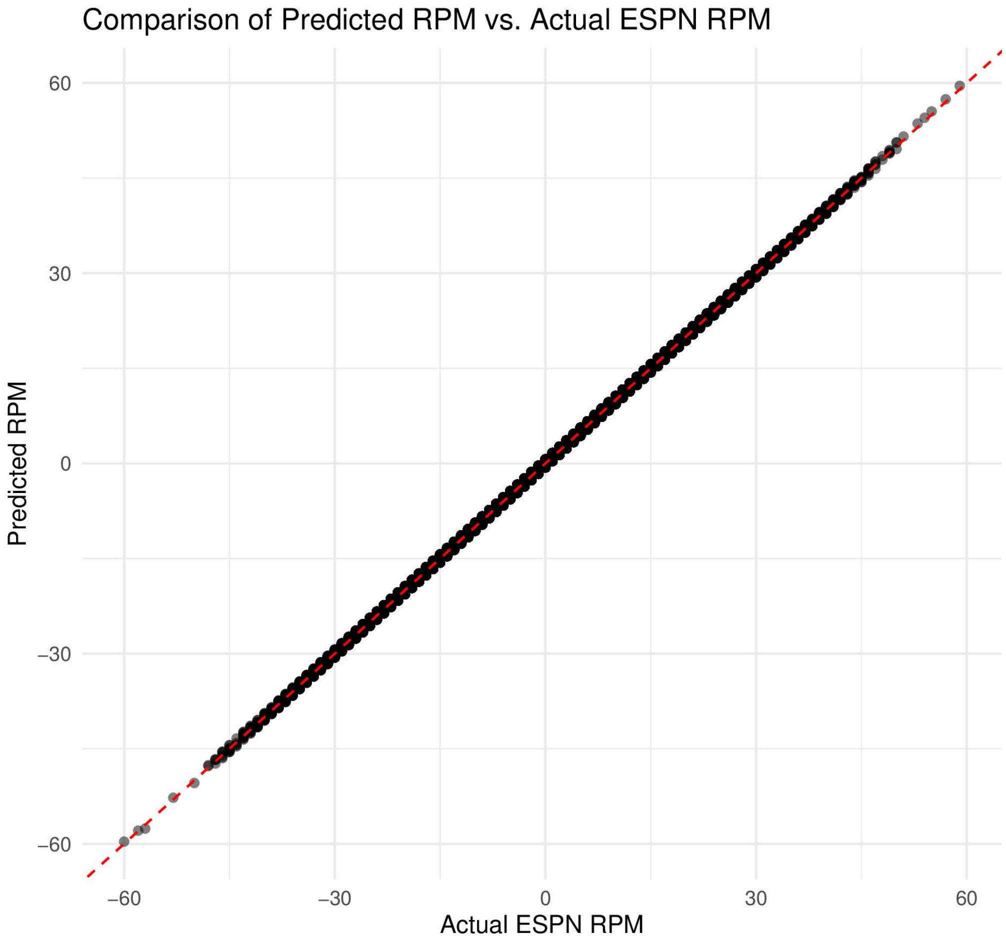

S4 Table: Distribution of Absence of Leaders

S4 Table: Observations Pre and Post Treatment by Absence Reason, Leader Type and Season

Reason	6th Foul						Injuries					
	Allstar		Captain		Both		Allstar		Captain		Both	
Season	Pre	Post	Pre	Post	Pre	Post	Pre	Post	Pre	Post	Pre	Post
2002	210175	1709	210466	1418	210791	1093	203572	8312	202097	9787	205814	6070
2003	434595	2921	433050	4466	435013	2503	418635	18881	421449	16067	423427	14089
2004	531083	4134	531500	3717	533008	2209	520310	14907	519744	15473	529615	5602
2005	555162	3765	552709	6218	556148	2779	536095	22832	537716	21211	548415	10512
2006	556928	3202	554070	6060	557630	2500	540975	19155	538326	21804	547619	12511
2007	558077	3291	556006	5362	558769	2599	539805	21563	544828	16540	549575	11793
2008	560568	3064	559517	4115	561160	2472	546897	16735	551892	11740	553990	9642
2009	558323	3806	556887	5242	559130	2999	554848	7281	541802	20327	555109	7020
2010	563065	3242	562124	4183	563799	2508	555061	11246	555250	11057	561736	4571
2011	564764	1819	562886	3697	565106	1477	550489	16094	552661	13922	557571	9012
2012	461206	1071	460716	1561	461323	954	436643	25634	449328	12949	454896	7381
2013	562605	1999	561796	2808	563029	1575	552047	12557	550143	14461	557897	6707
2014	578148	2621	576458	4311	578684	2085	557870	22899	561432	19337	568176	12593
2015	579305	1522	578427	2400	579368	1459	549608	31219	566570	14257	567526	13301
2016	584630	1583	584327	1886	584956	1257	574995	11218	580966	5247	577887	8326
2017	585294	1588	585224	1658	585716	1166	575959	10923	574033	12849	586209	673
2018	589526	1008	588825	1709	589551	983	577253	13281	580178	10356	585444	5090
2019	610624	2331	609720	3235	611271	1684	576141	36814	584246	28709	595727	17228
2020	537614	1305	536552	2367	538061	858	524419	14500	519135	19784	530813	8106
2021	529794	1276	529725	1345	530566	504	512083	18987	509196	21874	524790	6280

S5 Table: Distance of Two-Point Field Goals

S5 Table: Distance of Two-Point Field Goal Attempts

Dependent Variable:	Distance of Two Point Field Goal Attempts		
Model:	(1)	(2)	(3)
Both: Treatment × Post	-0.0370		
	(0.2988)		
Captain: Treatment × Post		-0.1456	
		(0.2807)	
All-Star: Treatment × Post			-0.7886
			(0.5478)
Home	-0.2784***	-0.2779***	-0.2781***
	(0.0144)	(0.0144)	(0.0144)
Score Difference	0.0024**	0.0024***	0.0024***
	(0.0009)	(0.0009)	(0.0009)
Period	-0.1521***	-0.1518***	-0.1535***
	(0.0104)	(0.0104)	(0.0104)
Fixed-effects			
Game	Yes	Yes	Yes
Player	Yes	Yes	Yes
Team	Yes	Yes	Yes
Opponent Team	Yes	Yes	Yes
Observations	1,760,377	1,760,377	1,760,377
R^2	0.141	0.141	0.141

Score Difference is *Team − Opponent*. Player-level clustered robust standard errors in parentheses. * $p < 0.1$; ** $p < 0.05$; *** $p < 0.01$.

S6 Table: Estimations for Away Teams

S6 Table: Score Difference, Game Result and Injury of Key Players (Away Teams)

Dependent Variable:	Score (1)	Score (2)	Score (3)	Result (1)	Result (2)	Result (3)
Injury of Both	−1.20***	−0.87***	−1.65***	−0.13***	−0.10**	−0.22***
	(0.20)	(0.19)	(0.25)	(0.03)	(0.03)	(0.05)
Injury of Only Captain	−1.28***	−0.09	−0.57*	−0.18***	−0.01	−0.08
	(0.17)	(0.15)	(0.24)	(0.03)	(0.03)	(0.05)
Injury of Only All-Star	0.64***	0.17	−0.12	0.07*	0.00	−0.05
	(0.19)	(0.17)	(0.28)	(0.03)	(0.03)	(0.05)
Opponent's Injury of Both	1.57***	1.23***	1.89***	0.19***	0.16***	0.27***
	(0.20)	(0.18)	(0.25)	(0.03)	(0.03)	(0.05)
Opponent's Injury of Only Captain	1.59***	0.30	0.17	0.20***	0.02	−0.01
	(0.17)	(0.16)	(0.25)	(0.03)	(0.03)	(0.05)
Opponent's Injury of Only All-Star	−0.15	0.27	0.54*	−0.04	0.01	0.08
	(0.19)	(0.17)	(0.28)	(0.03)	(0.03)	(0.05)
Play-off		−1.22***	−0.74*		−0.11*	−0.03
		(0.32)	(0.35)		(0.05)	(0.06)
Elo Difference		0.04***	−0.01***		0.01***	−0.00***
		(0.00)	(0.00)		(0.00)	(0.00)
Fixed-effects						
Team × Season	No	No	Yes	No	No	Yes
Opponent × Season	No	No	Yes	No	No	Yes
Observations	25,562	25,549	25,549	25,562	25,549	25,549
R^2	0.01	0.16	0.25			
Deviance				34368.13	31001.57	28727.36
Log Likelihood				−17184.06	−15500.79	−14363.68
Pseudo R^2				0.01	0.10	0.10

Game level clustered robust standard errors in parentheses. * $p < 0.1$; ** $p < 0.05$; *** $p < 0.01$.
